# A Case of Bilateral Central Retinal Artery Occlusion in a Post-COVID Rhino-Orbital-Cerebral Mucormycosis Patient

**DOI:** 10.7759/cureus.20062

**Published:** 2021-11-30

**Authors:** Abhishek Patnaik, Bharti Sharma, Reyaz Ahmad, Abhijit Kumar, Riti Chitrotpala, Minakshi Gupta

**Affiliations:** 1 Ophthalmology, Bokaro General Hospital, Bokaro Steel City, IND; 2 Ophthalmology, Tata Main Hospital, Jamshedpur, IND; 3 Neurology, Tata Main Hospital, Jamshedpur, IND; 4 Otolaryngology, Tata Main Hospital, Jamshedpur, IND; 5 Radiology, Tata Main Hospital, Jamshedpur, IND; 6 Microbiology, Tata Main Hospital, Jamshedpur, IND

**Keywords:** immunocompromised, post covid, mucormycosis, central retinal artery occlusion, rhino orbital cerebral mucormycosis

## Abstract

Central retinal artery occlusion (CRAO) is a multifactorial disease, where inflammation and hypercoagulability are the major risk factors. It is a rare occurrence in this geographical area in patients diagnosed with sinus mucormycosis infection, which has emerged as one of the most fulminant, opportunistic secondary infection during post-COVID era. We report a case of a bilateral CRAO, in a 66-year-old, post-COVID, diabetic patient. A complete eye examination followed by radiological imaging of brain, orbit, and paranasal sinuses were done. Multidisciplinary approach was contemplated to reach a diagnosis of bilateral rhino-orbital-cerebral mucormycosis (ROCM). Intravenous liposomal amphotericin-B injection was started as a part of systemic management and an aggressive sinus debridement of both sides with amphotericin-B wash was also done. Despite an early diagnosis and intervention, the patient succumbed to her illness. All post-COVID patients presenting with the complaints of blurring of vision should be meticulously examined for the presence of any retinal abnormality in both the eyes as this may be a manifestation of an underlying secondary fungal infection. Early diagnosis of ROCM and management will help in reducing complications.

## Introduction

It has been reported that about 10-30% of secondary infections occur in severely ill, hospitalized, COVID patients. Although viral and bacterial infections are more prevalent, fungal infections have also been found to be very common and virulent during the present pandemic [1,2]. Mucormycosis has emerged as a opportunistic, secondary fungal infection, which is found mostly in immunocompromised hosts. The fatality rate has increased substantially in immunocompromised conditions or whenever there is an involvement of orbit or intracranial structures [3,4]. It is most commonly caused by fungi of the order Mucorales. This fungal infection starts in the nasal or paranasal sinus mucosae and gradually spreads to the orbit and brain. Once inside the orbit, the disease is classified as rhino-orbital-cerebral mucormycosis (ROCM) [5,6].

Potentially serious ophthalmic problems associated with ROCM include central retinal artery occlusion (CRAO) and infarction of the optic nerve, endophthalmitis/panophthalmitis, globe erosion, and cavernous sinus involvement, which eventually leads to complete loss of vision. CRAO is a multifactorial disorder that is generally caused by a thromboembolic phenomenon. However, finding of a bilateral CRAO associated with ROCM is unusual in this part of geographical area of Jharkhand, and earlier, only 16-20% cases have been outlined [7-9]. From orbit, the fungus can spread to the brain through the cribriform plate and orbital apex, causing further complications and sometimes even death [10].

Herein, we report an unusual case of an immunocompromised patient with a post-COVID, poorly controlled diabetes status, presenting with ROCM and manifesting as a bilateral CRAO.

## Case presentation

**Figure 1:**
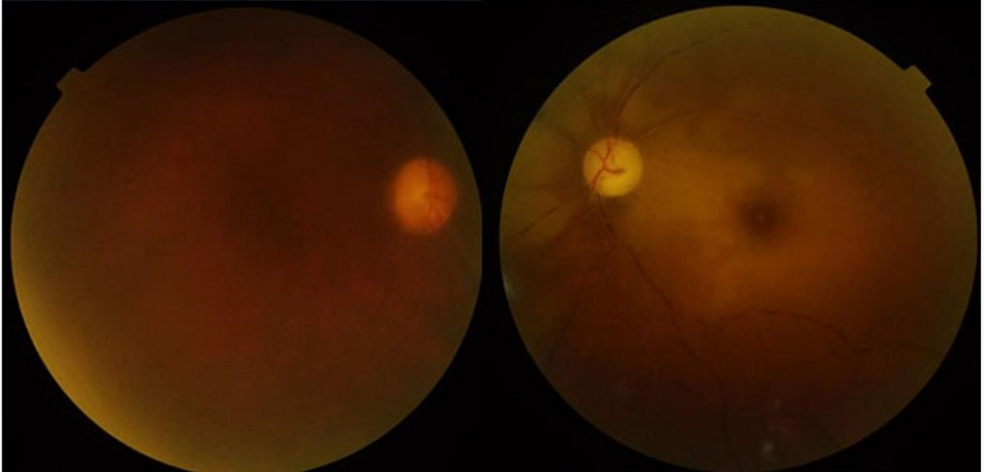
Bilateral central retinal artery occlusion.

A 66-year-old lady, only two days of her discharge from post-COVID ward, consulted the department of ophthalmology with complains of sudden painless loss of vision in her both eyes with drooping of upper eyelid in the right side. On presentation, she was conscious and afebrile but appeared disoriented. On examination, her best-corrected visual acuity was no light perception in both the eyes. There was a complete restriction of extraocular motility in all fields of gaze in both eyes with bilateral ptosis, which was more on the right than the left. Pupils of both the eyes were mid dilated and nonreactive to light reflex. Other anterior segment examination revealed that she was pseudophakic in both eyes and had a bilateral mild proptosis and periorbital puffiness of face. Fundus examination divulged the presence of a bilateral, pale optic disc with severe arterial narrowing. There was a retinal whitening in the posterior pole and loss of physiological macular reflex, suggestive of bilateral CRAO (Figure 1). Since the patient was sick and the blood glucose level and serum creatinine were highly deranged, a fundus fluorescein angiography could not be done.

She had a history of long-standing diabetes (more than 20 years) and had not got herself vaccinated against COVID-19. Having tested COVID positive, she was admitted in a COVID ward two weeks back and was treated with injectable antiviral drugs, steroids (injectable methylprednisolone), antibiotics, and high-flow oxygen support. After completing the treatment, she was discharged from the hospital, while maintaining an oxygen saturation of 92-95% in room air. Her hematological report during her stay in hospital revealed a hemoglobin A1c (HbA1c) of 12.7, raised random blood sugar of 490 mg/dl, and raised inflammatory markers such as C-reactive protein (10.05 mg/dl), serum ferritin (492.50 mg/dl), and erythrocyte sedimentation rate (ESR) (29 mm/h).

**Figure 2:**
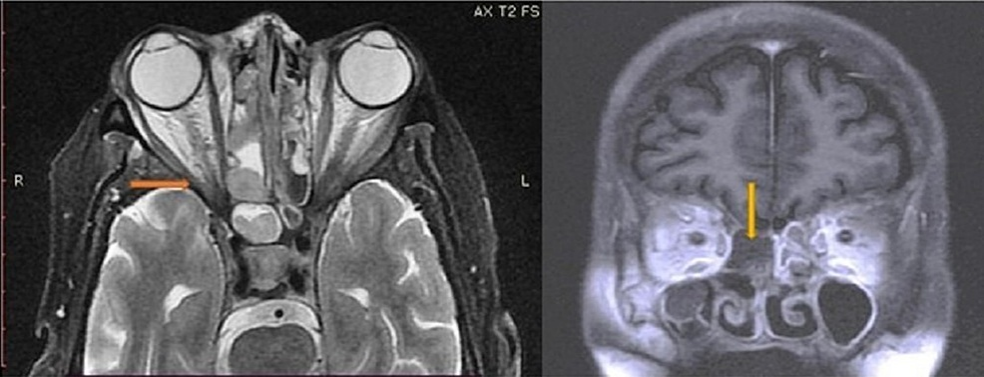
Radiology findings of orbits and sinuses. Orange arrow: periantral fat stranding, suggesting involvement of orbits; yellow arrow: black turbinate sign, denoted by lack of enhancement of the turbinates in post-contrast scan.

After an initial bedside eye examination, she was admitted in the medicine ward and was referred for an opinion to a neurologist and ear, nose, throat (ENT) specialist. A magnetic resonance imaging (MRI) of brain, orbit, and paranasal sinuses was requested after the neurologist consultation. Findings on T2-weighted MRI of the para-nasal sinuses showed a low attenuating mass in right maxillary, ethmoid, and sphenoid sinuses with surrounding mucosal thickening, consistent with a fungal infection. Post-contrast sequences showed nonenhancing angio-invasive fungal infection in the mucosa that gave the "black turbinate sign" appearance. Necrosed mucosa in the right nasal cavity and the right ethmoid sinuses was present, indicating acute invasive fungal sinusitis. Stranding of the periantral fat was seen in T1 and STIR/T2 fat-sat, suggesting involvement of the orbit (Figure 2).

**Figure 3:**
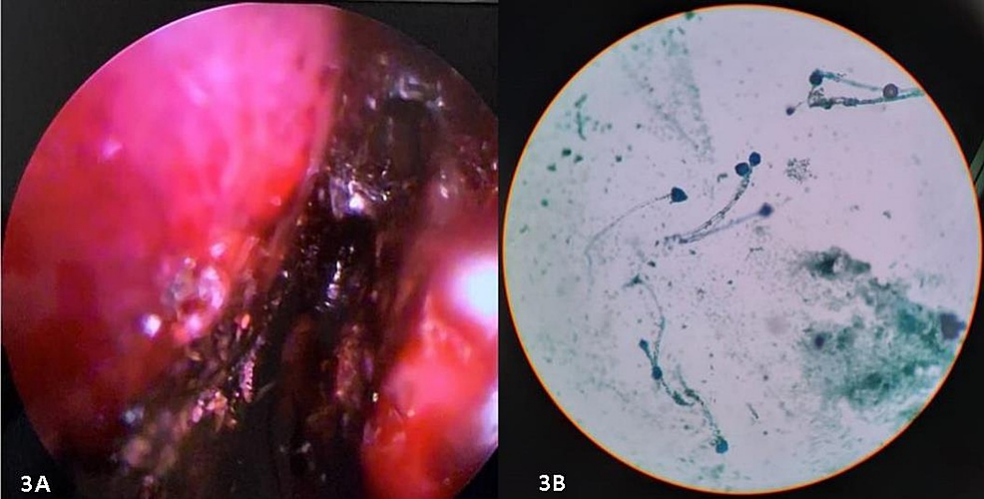
A) Nasal endoscopy picture; B) KOH mount picture.

An anterior rhinoscopy followed by a diagnostic nasal endoscopy (DNE) was performed by the ENT specialist. DNE showed bilateral extensive crusting and eschar formation in the nasal cavity (Figure 3A). Bilateral middle turbinates were insensitive to touch and necrotic. Eschar was noted along the lateral nasal wall and the posterior end of septum also. Multiple small biopsies were performed, and the specimen was sent for KOH mount and fungal culture (Figure 3B).

**Figure 4:**
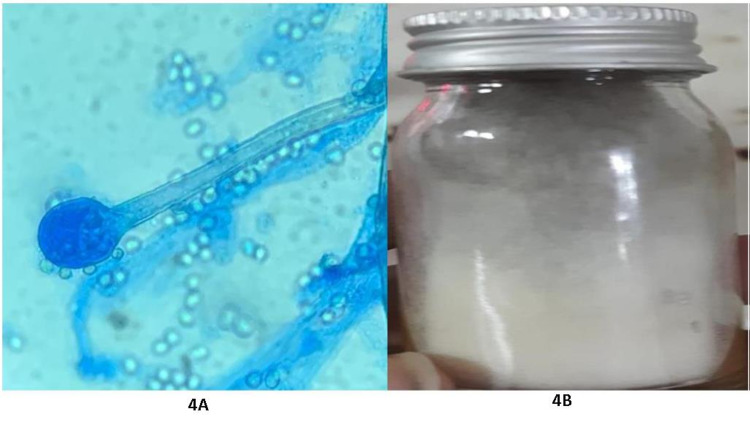
A) Broad, nonseptate, acute-angled hyaline hyphae; B) fungal colonies identified on LPCB mount. LPCB, Lacto Phenol Cotton Blue.

KOH mount reported the presence of broad, nonseptate, acute-angled hyaline hyphae (Figure 4A).

Based on a multispecialty corroboration, a diagnosis of a bilateral ROCM was made and she was started with intravenous liposomal amphotericin B (L-AMB) injection (50 mg vial), in a dose of 10 mg/kg/body weight. Under the consultation of the neurologist, every third day monitoring of her serum electrolytes (potassium and magnesium) and renal functions (serum creatinine) was planned, while maintaining her blood sugar with subcutaneous insulin. On the following day, eye examination revealed no perception of light and complete restriction of extraocular motility in her both eyes. Pupils were mid-dilated and did not respond to light stimulus. Hematological examination done in the ward revealed a deranged blood picture with an HbA1C level of 12.7 mg%, C-reactive protein 2.77 mg/dl, and an elevated serum potassium level of 6.3 mg/dl. Culture of the biopsy specimen on Sabouraud’s Dextrose Agar (Himedia M063) from the sinuses grew cotton-like fluffy colonies within a week. This later turned dark grayish. On performing Lacto Phenol Cotton Blue on colonies, fungus was identified as *Mucor* spp., and this established the diagnosis of a bilateral ROCM (Figure 4B).

An emergency endoscopic debridement under general anesthesia was planned for the patient. A bilateral endoscopic medial maxillectomy, bilateral ethmoidectomy with frontal recess clearance was done. Debridement was carried out till fresh bleeding was noted from all margins and the nasal cavity was packed before securing the case. Since the patient was very sick, orbit was spared; however, a transcutaneous amphotericin-B injection was planned for the next day. She received her second dose of L-AMB injection on the next day and insulin was titrated to keep the blood sugar level normal. On the first post-operative day, although she was responding to verbal commands, her eye condition still reported complete external ophthalmoplegia with a nonreacting pupil of both eyes indicating a cavernous sinus involvement in brain. Her visual perception showed no further improvement.

Her condition suddenly deteriorated toward the evening and she complained of breathlessness. There was a sudden fall in blood pressure, followed by cardiac arrest and she could not be resuscitated despite all efforts.

## Discussion

ROCM is the most common form of mucormycosis found in diabetic patients [5,8]. Mucormycetes are a group of distinctive mycoses that are ubiquitous, saprophytic fungi which can be cultured from the nasal mucosa of normal persons. Due to their angio-invasive nature, the fungi colonize in the sino-nasal mucosa and enter the orbit through the ethmoid and maxillary sinuses or via the nasolacrimal duct [11]. Ethmoid and maxillary sinuses are the most commonly infected sinuses in ROCM, followed by sphenoid and frontal sinuses [12].

CRAO is a habitual finding in hypercoagulable conditions and ascribed to embolization from ulcerated plaques of carotid artery or atherosclerotic occlusions of internal carotid artery [13]. A bilateral occurrence of CRAO, although rare, has been detected in conditions like atrial fibrillation, enteric fever, central nervous system lymphoma, and acquired immunodeficiency syndrome [14]. Due to its predilection for internal elastic lamina of blood vessels, the fungus directly infiltrates into the central retinal artery from the orbit, initiating a phenomenon of necrotizing vasculitis and thrombosis, which leads to ophthalmic ischemia and ophthalmoplegia [15]. CRAO is, thus, a rare occurrence in ROCM, and presentation of a bilateral CRAO in ROCM is even more rarely reported in the literature.

Uncontrolled diabetes is the most common predisposing factor for mucormycosis. This hyperglycemic status nourishes the fungal colonies by interfering with the neutrophilic phagocytosis and, thus, there is an increase in the availability of unbound iron in blood. The presence of excess ferritin in the blood of recently tested COVID-positive patients acts as an additional stimulant that buttresses the fungus causing extensive damage to the endothelial cells and extracellular matrix proteins lining the blood vessels [16]. Intracranial involvement also occurs by invasion through superior orbital fissure, ophthalmic vessels, cribriform plate, carotid artery, or via a perineural route [17]. In acute conditions, ROCM can cause vision loss, ptosis, diplopia, and external ophthalmoplegia, and, if left untreated, can progress to acute vision loss and even death. A state of hypercoagulability already exists in almost all COVID-19 patients due to a hyperinflammatory response to SARS-CoV-2 virus. The patient in discussion was an elderly woman who had recently tested COVID positive two weeks back and had a history of uncontrolled diabetes. The bilateral manifestation of CRAO in this patient was strongly indicative of a bilateral sinus involvement. Thus, a coexistence of a sinus disease with eye manifestations in a post-COVID, diabetic or any immunocompromised patient should make us circumspect about a fungal etiology. An agile, multispecialty connivance like ophthalmology, radiology, otolaryngology, neurology, and microbiology plays an important role in diagnosing and defining the extent of disease to commence a congruous management. 

The standard treatment for the early stages of ROCM is antifungal therapy and surgical debridement of the infected sinuses. Lipid formulations of amphotericin B (amphotericin B lipid complex and liposomal amphotericin B) are now the first drug of choice for treatment of ROCM. Liposomal encapsulation of amphotericin B enhances its penetration and efficacy. Till the infection is restricted to the sino-nasal or rhino-orbital area, high success rate with L-AMB and sino-nasal debridement is evident. So, a repeated surgical debridement of infected sites is required [17]. Once the infection is transformed to ROCM and has been demonstrating symptoms compatible with deep spread within the orbit, orbital exenteration is preferred. This remains the last expedient in the treatment regime to avoid the contiguous spread of the fungus to the vessels of the brain.

To abridge, although CRAO is a medical emergency that is usually known to be caused by thromboembolic episodes resulting from a hypercoagulable state such as that found in COVID-19 patients [18], the probability of mucormycosis causing a bilateral CRAO in a post-COVID patient due to direct infiltration into the ophthalmic artery is even more infrequently reported.

## Conclusions

Mucormycosis is a very lethal disease and heralds a poor prognosis because of the difficulty in its diagnosis and disseminating nature. A high degree of suspicion should always be kept in mind for the presence of a bilateral ROCM in the patients presenting with a CRAO as their first manifestation and particularly in those patients who have a history of a post-COVID or any immunocompromised status. Prompt inception of a systemic and surgical management after the diagnosis apprehends the intracranial spread of this fatal disease and thus improves the patient’s final outcome.
